# Improving ovarian cancer imaging with LHRH-NBs: an experimental study

**DOI:** 10.1007/s00404-016-4092-z

**Published:** 2016-04-13

**Authors:** Wenjuan Li, Lingping Zhang, Yuanfang Zhu, Jinyi Zhang, Li Shen, Shuying Huang, Shanyu Fang

**Affiliations:** 1 Department of Obstetrics and Gynaecology, Wuxi Maternal and Child Health Hospital, Wuxi, 214002 Jiangshu People’s Republic of China; 2 Department of Obstetrics and Gynaecology, Shenzhen Baoan Maternal and Child Health Hospital, Shenzhen, 518133 Guangdong People’s Republic of China; 3 Department of Obstetrics and Gynaecology, The First Affiliated Hospital of Nanchang University, Nanchang, 330006 Jiangxi People’s Republic of China

**Keywords:** In vivo imaging, Nanoliposomes, Ovarian cancer, Ultrasound contrast agent

## Abstract

**Purpose:**

Our previous study used freeze-drying and biotin–avidin binding methods and obtained nontargeted nanobubbles (N-NBs) and ovarian cancer-targeting nanobubbles (LHRH-NBs, luteinizing hormone-releasing hormone nanobubbles). Our study also identified the physical and chemical properties of these two contrast agents, and validated the targeting ability and underlying mechanisms of LHRH-NBs in vitro. The present study investigated the imaging of N-NBs and LHRH-NBs in nude mice and their binding with tissues.

**Methods:**

The nude mice models of xenografts were divided into three groups, N-NB, LHRH-NB, and SonoVue. These contrast agents were injected via the caudal vein to observe the imaging of ovarian cancer. Fluorescence microscope was used to observe the penetration of N-NBs and LHRH-NBs through the vascular endothelial gaps. Immunofluorescence was used to observe the penetration of N-NBs and LHRH-NBs through vascular endothelial gaps and binding to the tumor cells.

**Results:**

The imaging intensity and duration were not significantly different between N-NBs and LHRH-NBs. The imaging intensity in the N-NB and LHRH-NB groups was not significantly different compared with the SonoVue group; however, the imaging duration in the N-NB and LHRH-NB groups was significantly longer than in the SonoVue group (*P* < 0.001). Both N-NBs and LHRH-NBs penetrated through the vascular endothelial gaps. After penetrating through the vascular endothelial gapes, LHRH-NBs could target and bind to the tumor cells.

**Conclusions:**

N-NBs and LHRH-NBs are of good imaging effectiveness and relatively long imaging duration. LHRH-NB is a potent contrast agent for imaging ovarian cancer, while achieving targeted delivery of drugs to the site of ovarian cancer.

## Introduction

Ovarian cancer when compared to other gynecology-associated malignancies has the highest mortality rate [1]. As the onset of ovarian cancer is insidious, 80 % of the patients unknowingly get diagnosed as middle or late stage. International Federation of Gynecology and Obstetrics (FIGO) reported that the five-year survival rate is >80 % in women with stage I–IIa ovarian cancer, but was only <40 % in the ones with stage IIb–IV [2]. Therefore, early qualitative and location diagnosis of ovarian cancer is critical in improving the survival rate. The currently used CA125 and other serum markers including human epididymis protein 4 (HE4) that were reported in recent studies [3] could improve the diagnosis of ovarian cancers; however, their low specificity restricted their application in clinical practice [4–6].

As a real-time, noninvasive, and an accurate imaging method, ultrasonic contrast technology has wide applications in clinical practice [7]. With the development of ultrasonic contrast technology, scientists have been considering the possibility of applying noninvasive ultrasonic molecular imaging technology in the early identification of ovarian cancers. Targeting nanoscale liposome contrast agent is a critical factor in ultrasonic molecular imaging. Most of the currently used contrast agents have a relatively large particle size (diameter of about 2–8 μm) and thus could not penetrate the blood vessels. Therefore, these agents could only display the vessel pool and hence relatively poor imaging of tissues. Nanobubble contrast agents could easily penetrate the blood vessels and aggregate at the imaging sites [8, 9]. Therefore, aggregation of these contrast agents with extremely low echo at the tissues outside the blood vessels could result in distinct enhancement signals at the target regions, thereby improving the imaging of the target regions while maintaining low background interference. In addition, the small particle size also reduced the phagocytosis by the immune system to some extent, which increased the circulation time of the agents in the blood with improved stability, and thus improved quality of the image. However, most of the nanobubbles are nonspecific and could not actively target to the lesion tissues with specific affinity; in addition, many nanobubbles could also nonspecifically bind to the hepatic sinusoid, splenic sinus, or vascular endothelial system after intravenous injection, and thus could not aggregate at the target tissues effectively [10]. Researchers have recently shown interest in sustained large-scale nanobubble aggregation at the target regions, which has led to their increased focus on achieving long-term imaging. With the advancements in the surface antigens of the tumor tissues, the researchers linked the specific antibodies or ligands to the surface of the nanobubbles to construct tumor-targeting nanobubble contrast agents, thereby providing a new idea in tumor-targeting ultrasound imaging. Previous studies have shown that luteinizing hormone-releasing hormone (LHRH) receptor is overexpressed in ovarian cancer cells; however, the expression in normal ovarian tissues could hardly be detected [11, 12].

In light of these findings, in our previous studies [13], we chose film-forming materials as the shell, and fluothane as the core to obtain nontargeting nanobubbles (N-NBs) via freeze-drying method, and then, the LHRH antibody was linked to the surface of the nanobubble via biotin–avidin binding method to prepare ovarian cancer-targeting nanobubbles (LHRH-NBs). Both these nanobubbles have small particle sizes (295–468 and 369–618 nm, respectively) and high stability. LHRH-NBs could specifically and efficiently bind to human ovarian cancer OVCAR-3 cells in vitro. Although the in vitro studies have shown that LHRH-NBs have high targeting features, the in vivo imaging profiles using LHRH-NBs are still unclear. In the present study, both these nanobubble contrast agents that have already been successfully prepared by us were used for imaging of nude mice models of xenografts to investigate the in vivo imaging efficiencies and their binding to tumor cells.

## Materials and methods

### Materials

Dipalmitoyl phosphatidylcholine (DPPC), distearoyl phosphatidylethanolamine (DSPE), and biotinylated dipalmitoyl phosphatidylethanolamine (DSPE-PEG2000-Biotin) were obtained from Avanti Company (Alabama, USA). Biotinylated LHRH antibody and FITC-labeled goat anti-rabbit immunoglobulin (Ig) G were from Beijing BiossBiological Technology Co. LTD. (Beijing, China). Perfluorinated propane (C_3_F_8_) was purchased from the Tianjin Research Institute of Physical and Chemical Engineering of Nuclear Industry (Tianjin, China). OVCAR-3 cells were obtained from Huiying Biological Technology Co. LTD. (Shanghai, China). Avidin and McCoys 5A culture medium were obtained from Boster Biological Technology Co. LTD. (Wuhan, China). Blocking serum, 4,6-diamidino-2-phenylindole (DAPI), and cell membrane red fluorescent probe (DiI) were from Beyotime Biological Technology Co. LTD. (Shanghai, China). SonoVue was obtained from Bracco Imaging B.V. Company (Monroe, Switzerland). BALB/c nude mice were from the Experimental Animal Center of Chongqing Medical University, and the protocols in this study were approved by the Ethics Committee of Chongqing Medical University. The DFY ultrasound image quantitative analysis of diagnostic equipment was provided by Institute of Ultrasound Imaging, Chongqing Medical University. Philips iU 22 was from Philips (Amsterdam, the Netherlands). CKX41 inverted fluorescence microscope was from Olympus Company (Tokyo, Japan).

### Cell culture

The human ovarian cancer cells (OVCAR-3) were cultured in McCoys 5A medium containing 10 % heat-inactivated fetal calf serum at 37 °C and incubated in 5 %CO_2_. Cells were split every 2–3 days, and experimental cells were in log-growth phase.

### In vitro experiment

We have successfully prepared nanoscale liposome microbubbles, namely N-NBs and LHRH-NBs, in our previous studies [13] via freeze-drying and biotin–avidin binding methods. The physical and chemical properties of these two microbubbles were explored, and the targeting ability and underlying mechanisms were investigated in vitro.

### Induction of mice models of ovarian cancer xenografts

Thirty female BALB/c nude mice aged 4–6 weeks were obtained. OVCAR-3 cells in the logarithmic growth phase were collected to obtain cell suspension with a density of 1 × 10^7^/ml. Then, 0.2 ml of the suspension was subcutaneously injected into the right hip of the mice. After the tumor reached a size of 1.0 cm, the following experiments were performed.

### Imaging ovarian cancer tissues with N-NBs, LHRH-NBs, and SonoVue

The mice were anesthetized with 10 % chloral hydrate. The mice were then fixed on the operating table, and body temperature was maintained using a heater. N-NBs (7.0 × 10^6^) were resuspended in 200 μl phosphate-buffered saline (PBS), and then, the suspension was injected via the caudal vein. The ultrasound probe was placed at the largest transverse section of the tumor, the imaging of the tumor was dynamically observed, and the images were preserved. Equal volume of the other two contrast agents, namely LHRH-NBs and SonoVue, were also injected via the caudal vein, and the data were recorded under the nontargeting contrast agent model. DFY ultrasound image quantitative analysis of diagnostic equipment was used to analyze the images.

### Observing penetration of nanobubbles through vascular endothelial gaps with fluorescence microscope

The nude mice were injected 250 μl of DiI-labeled N-NBs or LHRH-NBs via the caudal vein. The tumors were collected after imaging, and rapid-frozen slices of 5 μm thick were obtained. DAPI was used to stain the cellular nuclei before the observation to further clarify the distribution of the nanobubbles. The distribution of the nanobubbles was observed with CKX41 inverted fluorescence microscope. The excitation wavelength was 550 and 340 nm, and the emission wavelength was 560 and 490 nm for DiI and DAPI, respectively.

### Observing the binding of nanobubbles to tumor cells with immunofluorescence

LHRH-NB was injected into the tumor-bearing nude mice via the caudal vein, and the tumor was harvested after imaging. Rapid-frozen slices, 5 μm thick, were prepared immediately and washed with PBS thrice for 3 min each time, and then, FITC-labeled goat anti-rabbit IgG (1:100) was added. The slices were incubated for 30 min in dark, and the fluorescence was observed. Same procedures were applied for N-NBs to obtain the slices and observe the fluorescence. For the blank control, the tumor tissues were harvested without injection of contrast agent, and then, the rapid-frozen slices, 5 μm thick, were prepared. These slices were also washed with PBS thrice for 3 min each time and blocked with blocking serum for 30 min at 37 °C, and then, primary LHRH antibody (1:100) was added and incubated at 4 °C overnight. The slices were then placed at 37 °C for 30 min and washed with PBS thrice for 5 min each time. FITC-labeled goat anti-rabbit IgG (1:100) was then added and incubated for 30 min in dark, and fluorescence microscope was used to observe the fluorescence. The absorption wavelength of FITC was 490 nm, and the emission wavelength was 520 nm.

### Statistical analysis

SPSS 19.0 software was used for statistical analysis. Quantitative data are described as mean ± SD. Analysis of variance was used for comparisons between different groups. *P* < 0.05 was considered statistically significant.

## Results

### Particle size and distribution of N-NBs and LHRH-NBs in vitro

**Fig. 1:**
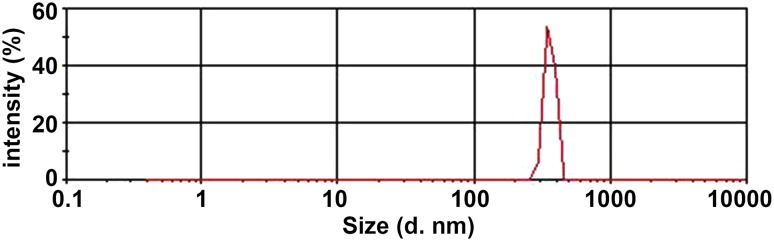
Physicochemical properties of N-NBs. The particle size ranged from 295 to 468 nm with a mean of 360 nm. N-NBs, nontargeted nanobubbles

**Fig. 2:**
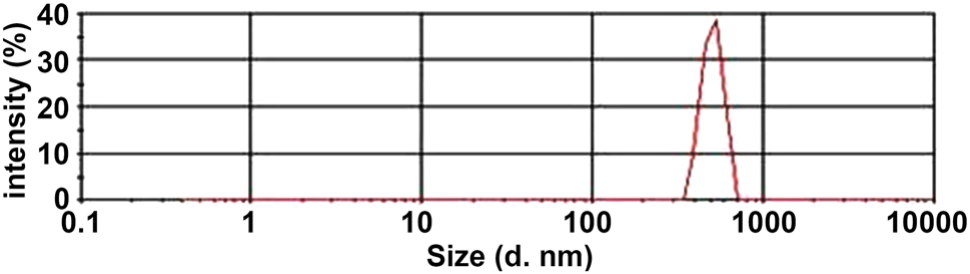
Physicochemical properties of LHRH-NBs. The particle size ranged from 369 to 618 nm with a mean of 508 nm. LHRH-NBs, ovarian cancer-targeting nanobubbles

In our previous studies [13], the two liposome contrast agents, namely N-NBs and LHRH-NBs, appeared as oyster white suspension. When observed under microscope at 400× magnitude, both the contrast agents were round, homogeneously distributed, well scattered, and with no aggregation. The particle size of N-NBs and LHRH-NBs was 295–468 and 369–618 nm, respectively (Figs. 1, 2).

### In vitro targeting and blocking of LHRH-NBs

**Fig. 3:**
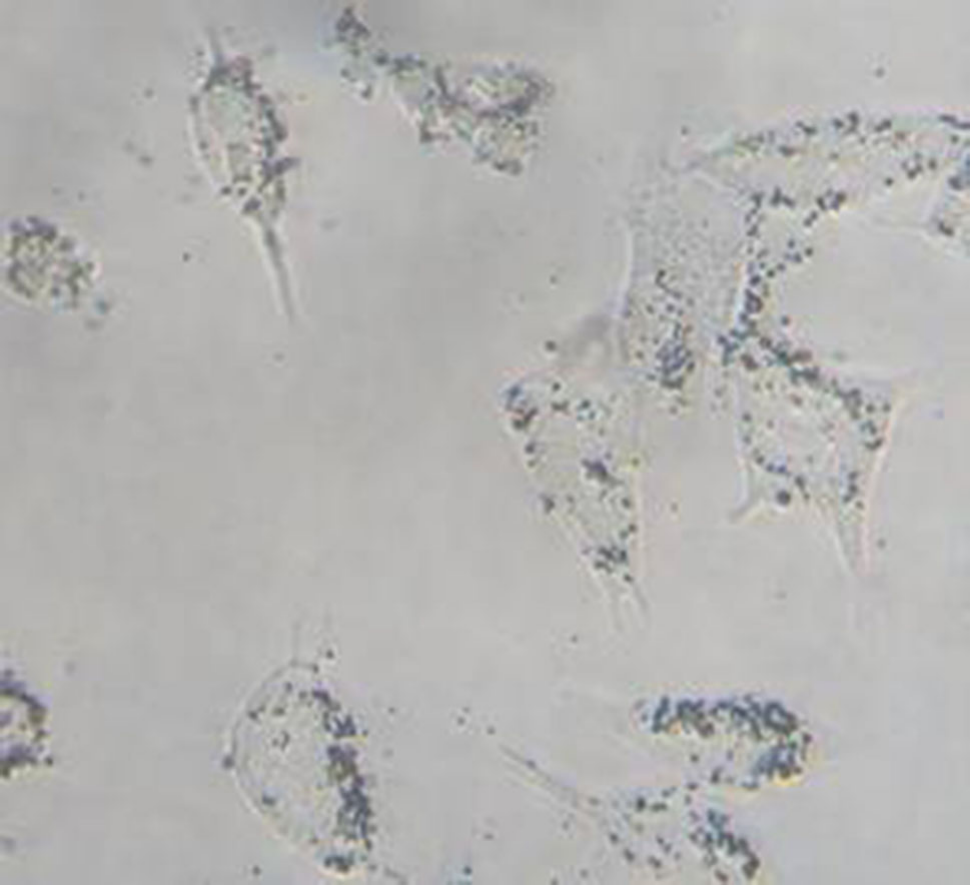
Light microscopy image of OVCAR-3 cells incubated with LHRH-NBs (magnification ×200). LHRH-NBs adhered to the cells and formed a rosette-like structure. LHRH-NBs, ovarian cancer-targeting nanobubbles; OVCAR-3, human ovarian cancer cells

**Fig. 4:**
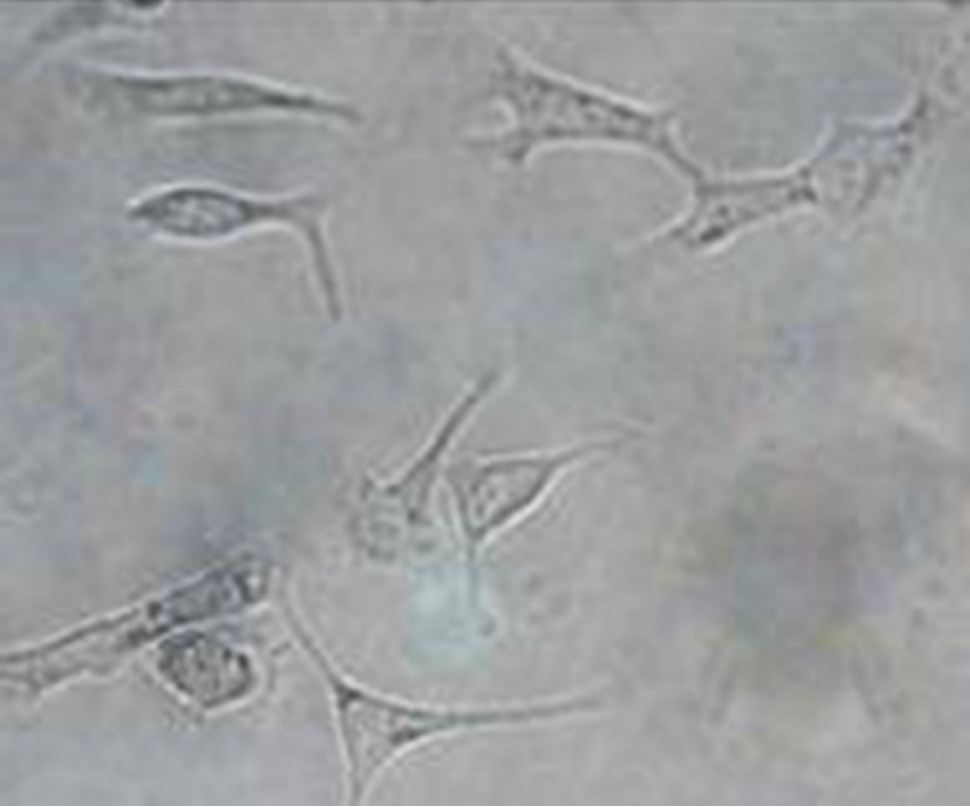
Light microscopy image of OVCAR-3 cells pre-incubated with LHRH antibody prior to treatment with LHRH-NBs. No binding of the microbubbles to OVCAR-3 cells was observed, and no rosette formation was detected (magnification ×200). LHRH-NBs, ovarian cancer-targeting nanobubbles; OVCAR-3, human ovarian cancer cells

Our previous studies [13] demonstrated that LHRH-NBs could target and bind to OVCAR-3 cells (Fig. 3), which could be blocked by pre-treatment of primary LHRH antibody (Fig. 4).

### Imaging of the ovarian cancer tissues using N-NBs, LHRH-NBs, and SonoVue

**Fig. 5:**
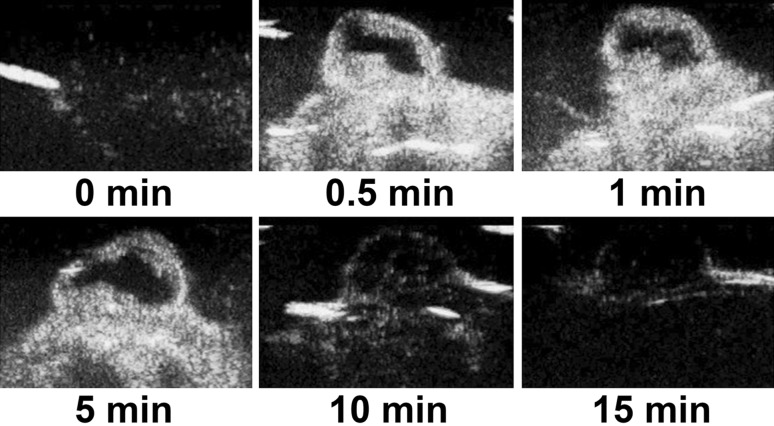
Dynamic imaging of the ovarian cancer with time using N-NBs (0, 0.5, 1, 5, 10, 15 min). N-NBs, nontargeted nanobubbles

**Fig. 6:**
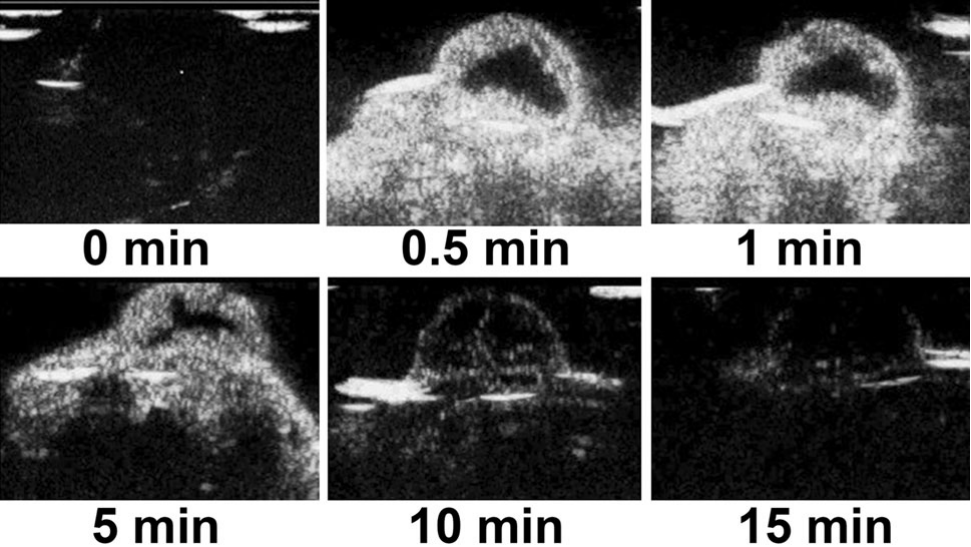
Dynamic imaging of the ovarian cancer with time using LHRH-NBs (0, 0.5, 1, 5, 10, 15 min). LHRH-NBs, ovarian cancer-targeting nanobubbles

**Fig. 7:**
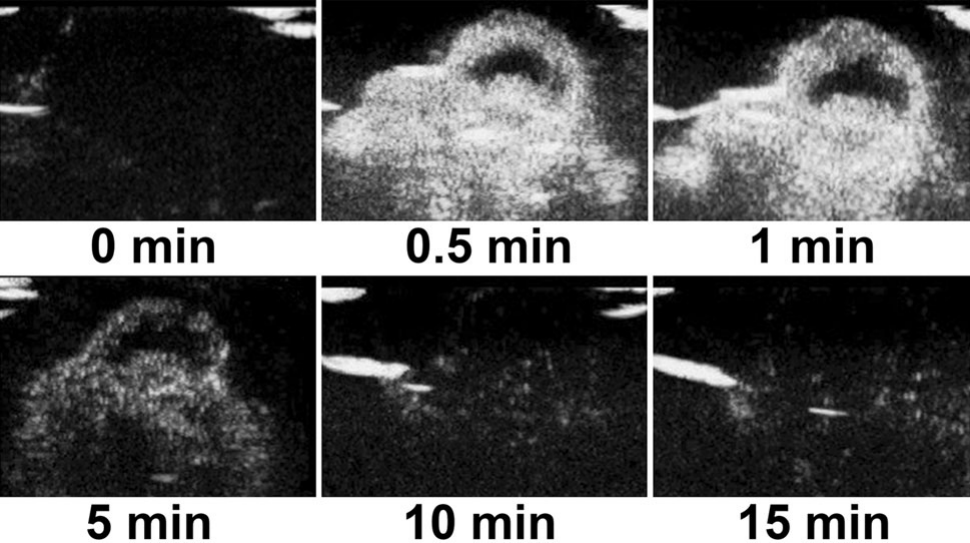
Dynamic imaging of the ovarian cancer with time using SonoVue (0, 0.5, 1, 5, 10, 15 min)

**Fig. 8:**
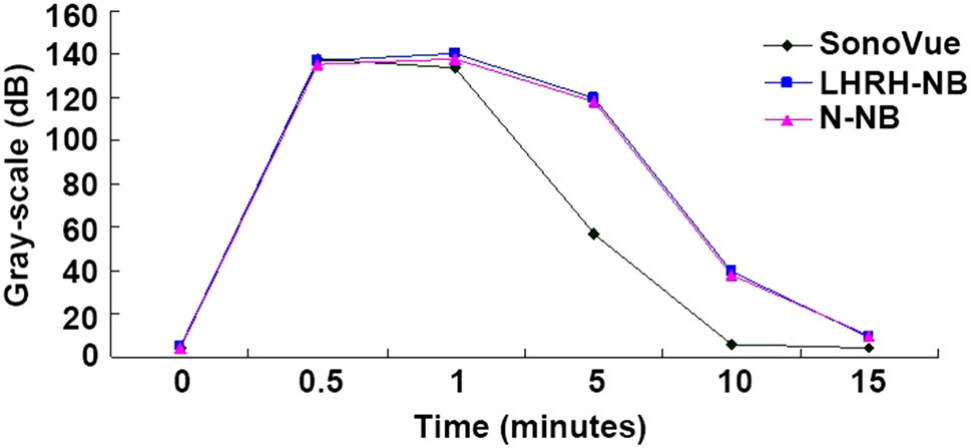
Decrease in gray scale with time is slower in N-NB and LHRH-NB groups than in the SonoVue group

No tumor-bearing nude mice died during the experiment. The dynamic changes in the three contrast agents, namely N-NBs, LHRH-NBs, and SonoVue, with time (0, 0.5, 1, 5, 10, and 15 min) are shown in Figs. 5, 6, and 7. The imaging intensity was not significantly different between these three contrast agents (*P* > 0.05). However, the imaging duration was longer in N-NBs and LHRH-NBs than SonoVue (Table 1) (*P* < 0.001). Figure 8 shows that the decrease in the imaging intensity with time was slower in N-NBs and LHRH-NBs than SonoVue.

**Table 1:** Imaging duration of three contrast agents

Group	Imaging duration (s)										Mean ± SD
	1	2	3	4	5	6	7	8	9	10	
N-NB group	1302	1201	1218	1304	1290	1253	1308	1243	1279	1295	1269.3 ± 38.30 i
LHRH-NB group	1309	1312	1216	1318	1212	1290	1274	1257	1318	1273	1277.90 ± 39.64 ii
SonoVue group	313	321	319	331	324	327	312	301	302	318	316.80 ± 9.93 iii

i versus iii, *P* < 0.001; ii versus iii, *P* < 0.001; i versus ii, *P* > 0.05

### Penetration of nanobubbles through the vascular endothelial gaps

**Fig. 9:**
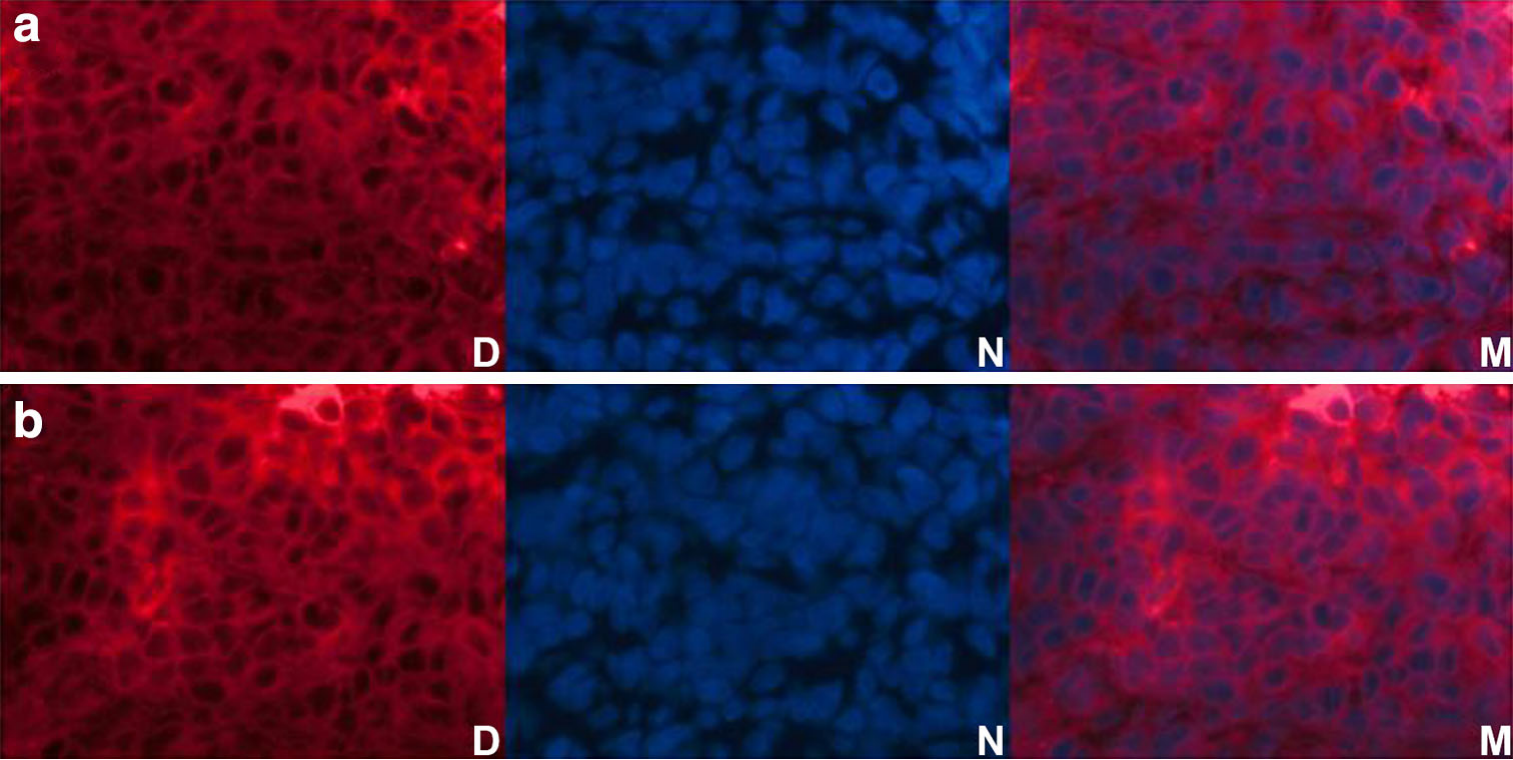
Distribution of the DiI-labeled nanobubbles in the tissues. The DiI-labeled N-NBs and LHRH-NBs are distributed in the tissue spaces, suggesting that both N-NBs and LHRH-NBs can penetrate through the vascular endothelial gaps. **a** (the *upper line*) Distribution of DiI-labeled N-NBs in the tumor tissues. **b** (the *bottom line*) Distribution of DiI-labeled LHRH-NBs in the tumor tissues. *D* DiI, *N* nucleus, *M* merge

Figure 9 shows the distributions of the DiI-labeled N-NBs and LHRH-NBs in tumor tissues. The nanobubbles could penetrate through the vascular endothelial gaps and fill the tissue stroma.

### Binding of nanobubbles with tumor cells after penetrating through the vascular endothelial gaps

**Fig. 10:**
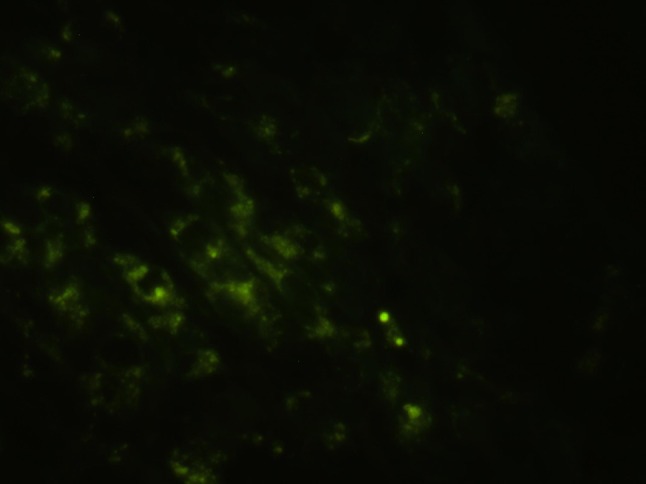
Fluorescence of the binding of LHRH-NBs with the tumor cells. LHRH-NBs penetrate through the vascular endothelial gaps and bind to the receptors on the tumor cell surface. Therefore, LHRH-NBs could bind to the tumor cells, and the fluorescence is scattered around the cells

**Fig. 11:**
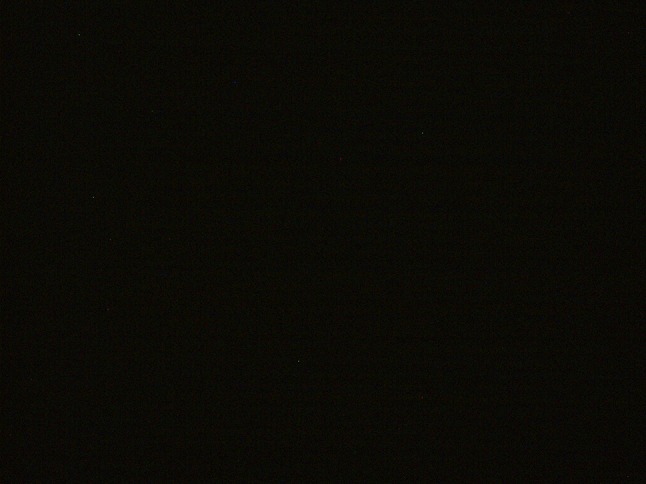
No fluorescence was found in the N-NB group. Although N-NBs could penetrate through the vascular endothelial gaps, it cannot bind to LHRH receptors on the tumor cell surface; therefore, it could be washed by PBS. Therefore, no fluorescence was found in the N-NB group

**Fig. 12:**
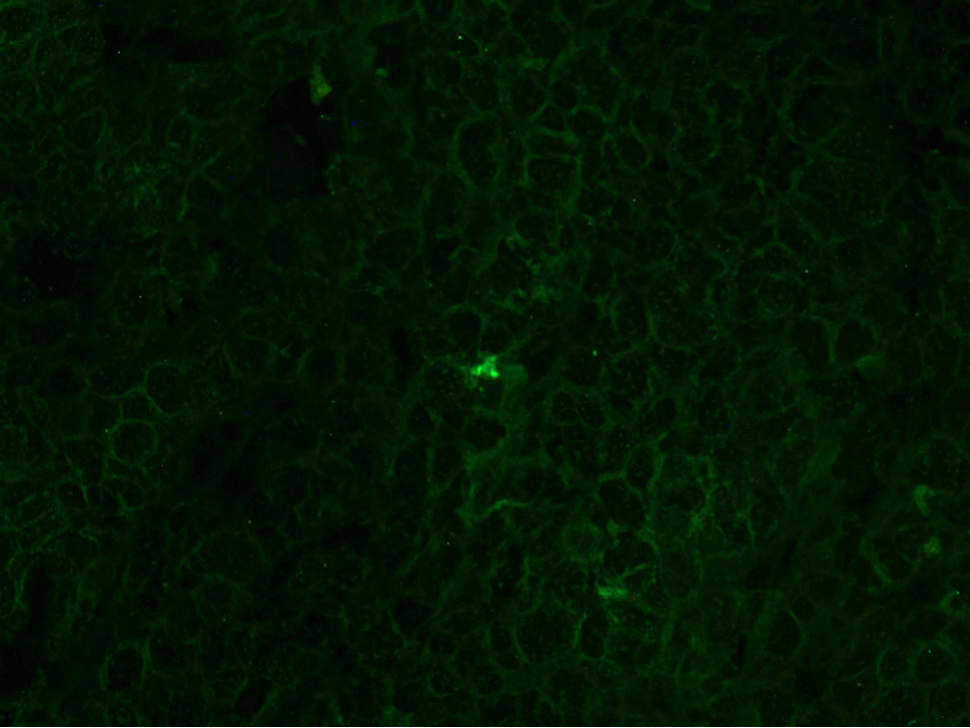
High intensity of fluorescence in the blank control group. Primary LHRH antibody (1:100) was added and incubated with the tumor tissues at 4 °C overnight, Therefore, large amount of fluorescence around the cells was found in the blank control group

The results of immunofluorescence examinations in the LHRH-NB group are shown in Fig. 10. LHRH-NBs could penetrate through the vascular endothelial gaps and bind to the receptors on the tumor cell surface. Therefore, LHRH-NBs could bind to the tumor cells, and the fluorescence is scattered around the cells. However, in the N-NB group, no fluorescence was found (Fig. 11). Large amount of fluorescence around the cells was found in the blank control group (Fig. 12).

## Discussion

With the development of ultrasonic contrast technology, the molecular imaging technology has shown great advantages in the early diagnosis of diseases. The evaluation of the imaging effectiveness of a contrast agent is mainly dependent on the following aspects: imaging intensity, imaging duration, and background interference. Micron-sized contrast agents are with relatively large particle size and gas contents, and the imaging intensity should be better than nanoscale contrast agents theoretically. However, the findings of the present study showed that the imaging effectiveness was not significantly different between the two nanoscale contrast agents (N-NBs and LHRH-NBs) and the micron-sized contrast agent SonoVue. The reasons could be as follows: (1) Nanoscale contrast agents visualize not only the vessel pool but also the tissues, and the latter could compensate the weakness of the nanoscale contrast agents in the intravascular imaging, and (2) when circulating in the body of the nude mice, the body temperature of the mice was higher than room temperature, the size of the nanoscale contrast agents could increase in a relatively high temperature, and some of them could even increase to almost the size of micron-sized contrast agents. Deshpande et al. [14] have shown that nanobubbles could merge with larger bubbles in the tissue spaces under ultrasound. These could be the reasons for comparable imaging effectiveness between the nanoscale and micron-sized contrast agents. However, the imaging duration in the N-NB and LHRH-NB groups was significantly longer than in the SonoVue group, and the reasons could be as follows: (1) The particle size of SonoVue is relatively large. Previous studies [15, 16] have already shown that the effectiveness of the enhancement is associated with the concentration and size of the contrast agent, as well as the frequency of the ultrasound. Larger-sized microbubble is easier to rupture in a certain extent and is easier to result in cavitation effect; (2) the relatively large-sized bubble could be easily eliminated by macrophages, thus the circulating time is relatively short; (3) long circulating PEG materials were used when preparing N-NBs and LHRH-NBs, which could greatly reduce the rapid elimination of the nanobubbles by the phagocytic system [17]; and (4) the nanobubbles could reside in the tissue spaces after penetrating the blood vessels, and thus, the hemodynamic effects are relatively low. The combined effects of these factors finally resulted in significantly longer circulating time of N-NBs and LHRH-NBs than SonoVue, and thus increased the imaging duration, which is more suitable for imaging in clinical practice.

Both N-NBs and LHRH-NBs are nanoscale contrast agents, but which one has better imaging effectiveness? We hypothesized that after reaching the target regions, LHRH-NBs, the contrast agent with extremely high penetration ability, could bind to the LHRH receptor expressed on the surface of the ovarian cancer cells through the LHRH antibody on the surface of the LHRH-NBs, and actively adhere to the tumor cells for a long time and aggregate in the tissue cells. Our previous studies have demonstrated the in vitro targeting ability of LHRH-NBs, which was confirmed by results from the fluorescence microscope and immunofluorescence examinations in the present study. The aggregation imaging evidently increased the signals in the targeted tumor regions and also maintained low background noise. Therefore, compared with N-NBs, LHRH-NBs theoretically have higher imaging intensity and longer imaging duration, and thus be a promising contrast agent in molecular imaging of ovarian cancer. However, the findings of the present study showed that the imaging effectiveness was not significantly different between the N-NB and LHRH-NB groups, and the reasons could be as follows: (1) both N-NBs and LHRH-NBs are identically affected by the hemodynamics in the blood vessels, and thus, the effects on the intravascular imaging are negligible; (2) the binding of LHRH-NBs to the tumor cells requires some time, while some bubbles could rupture even before binding to the tissues, thus the binding rate of LHRH-NBs to the tissue cells could be limited; and (3) the mobility of the tissue liquid is relatively low, thus N-NBs could remain in the tissue spaces even without actively binding to the cells and thus imaging the tissues. These factors finally caused the numbers of the two nanobubbles in the imaging of the tissues to be not significantly different; thus, compared with N-NB group, the imaging intensity and duration in the LHRH-NB group was not significantly different.

Although LHRH-NBs manifested no outstanding imaging advantages compared with N-NBs, fluorescence microscopy and immunofluorescence examinations in the present study showed that LHRH-NBs could penetrate through the endothelial gaps, followed by targeting and binding to ovarian cancer cells. Therefore, it is possible to use these nanoscale microbubbles as a vehicle to load the drugs and thus perform targeted therapy for ovarian cancer. We hypothesized that after penetrating through the newly developed tumor blood vessels with relatively loose endothelial spaces [18], the LHRH-NB loading with the drugs could then remain in the ovarian cancer tissues and cell surface for a long time. Therefore, active monitoring, localization, and controlled release of the ultrasonic energy to rupture the drug-loading microbubbles during the ultrasound imaging, high concentrations of the drugs could release in the targeted ovarian cancer regions; however, if no ultrasound is applied in the nontargeted regions, none or only a few drug-bearing microbubbles will rupture, and thus the drug concentration will be very low. The rupture of the microbubbles from repeated ultrasound in the targeted tumor regions will result in cavitation effect and mechanical effect [19], which will increase the vascular permeability and width of the endothelial cell gaps; thus, the drug-loading microbubbles could continuously penetrate through the damaged blood vessels to circulate and reperfuse, and result in targeted therapy with the drugs in high efficiency. Therefore, using minimum dose of the drugs could obtain the best treatment efficacy, and thus reduced the adverse effects of the chemotherapy and decreased the incidence of drug resistance. Milgroom et al. [20] connected Herceptin to silica nanoparticles and obtained nanoscale Herceptin-targeting ultrasound contrast agent. In vitro study demonstrated that this targeting nanoscale ultrasound contrast agent could achieve targeted binding to breast cancer cell HER-2 cells and exert cell-killing effects to some extent, and it also achieved successful imaging in vitro. The targeted drug delivery and localization release strategy based on the rupture of the drug-loading contrast agents with ultrasound provides a new direction for the targeted treatment for ovarian cancer with high efficiency.
